# Safety and Risks of Antihypertensive Medications During Breastfeeding: A Review of Current Guidelines

**DOI:** 10.3390/jcm14113722

**Published:** 2025-05-26

**Authors:** Emilia Piotrkowicz, Piotr Skrzypczyk, Aleksander Prejbisz, Piotr Dobrowolski, Maciej Gawlak, Przemysław Kosiński

**Affiliations:** 1 Student Scientific Group, Department of Pediatrics and Nephrology, Medical University of Warsaw, 02-091 Warsaw, Poland; em.piotrkowicz@gmail.com; 2 Department of Pediatrics and Nephrology, Medical University of Warsaw, 02-091 Warsaw, Poland; 3 Department of Epidemiology, Cardiovascular Prevention and Health Promotion, National Institute of Cardiology, 04-628 Warsaw, Poland; aprejbisz@ikard.pl (A.P.); pdobrowolski@ikard.pl (P.D.); 4 Department of Obstetrics, Perinatology, Gynecology and Reproductive Medicine, Medical University of Warsaw, 02-091 Warsaw, Polandprzemyslaw.kosinski@wum.edu.pl (P.K.)

**Keywords:** arterial hypertension, breastfeeding, antihypertensive medications

## Abstract

Hypertension disorders of pregnancy affect almost 10% of pregnancies. Most hypertensive disorders associated with pregnancy, including chronic hypertension and gestational hypertension, often persist into the postpartum period. Thus, many breastfeeding mothers require ongoing antihypertensive treatment with antihypertensive medications while nursing. This highlights the importance of understanding the efficacy, safety, and potential adverse effects of antihypertensive therapy in breastfeeding mothers. Unfortunately, research in this area is limited, and references in clinical guidelines remain sparse. Our review aims to provide a comprehensive summary of the current knowledge on antihypertensive medications during breastfeeding, drawing from available research and evidence-based guidelines. This article discusses all groups of antihypertensive drugs, presenting societies’ recommendations and available clinical data. Based on the available literature, calcium channel blockers (extended-release nifedipine as the first choice) and beta-blockers (labetalol, metoprolol) appear to be the drugs of choice. Our review highlights the need for further research to evaluate the long-term safety of antihypertensive medications during breastfeeding, improve clinical guidelines, and ensure optimal treatment for nursing mothers.

## 1. Introduction

Hypertension (HT) disorders of pregnancy affect almost 10% of pregnancies worldwide and remain the leading cause of maternal, fetal, and neonatal morbidity and mortality [1]. According to all compared guidelines, hypertension in pregnancy is diagnosed when systolic blood pressure is ≥140 mmHg and/or diastolic blood pressure is ≥90 mmHg. The risks associated with arterial hypertension in pregnancy include low birth weight, preterm birth, placental abruption, and prolonged high-level neonatal care [2,3]. HT during pregnancy is categorized into two main groups: chronic hypertension and hypertensive disorders of pregnancy, which include pregnancy-induced hypertension and pre-eclampsia [4].

Maternal blood pressure typically drops immediately after delivery but may rise again, reaching a peak around 3–6 days postpartum. Given the physiological changes in blood pressure during the postnatal period, it is important to maintain close monitoring and continue antihypertensive treatment during the first week postpartum to avoid unnecessary or overly aggressive treatment [4]. Most hypertensive disorders associated with pregnancy, including chronic hypertension and gestational hypertension, often persist into the postpartum period. As a result, many breastfeeding mothers will require ongoing treatment with antihypertensive medications while nursing. A systematic review and meta-analysis by Rameez et al. indicates that breastfeeding for more than 12 months is linked to a lower risk of developing hypertension later in a woman’s life, compared to breastfeeding for less than 12 months [5]. This highlights the need to better understand the efficacy, safety, and potential adverse effects of antihypertensive drugs in breastfeeding mothers. However, research in this area remains limited, and clinical guidelines on recommended medications are still scarce.

The primary aim of our review is to provide a comprehensive summary of current guidelines on the use of antihypertensive medications during breastfeeding. The secondary objective is to examine the latest research evaluating the potential risks of antihypertensive treatment for both lactating mothers and their infants.

## 2. Methods

The authors conducted a non-systematic review in the following electronic sources: MEDLINE, EMBASE, SCOPUS, and GOOGLE SCHOLAR. We used the following terms in our search: (“HYPERTENSION”) AND (“BREASTFEEDING OR POSTPARTUM OR PREGNANCY”) AND (“RECOMMENDATIONS” OR “GUIDELINES”) published from 2000 to May 2025, achieving 1985 records. When two guidelines from the same authors or organizations were found, only the most recent one was included in the analysis. We excluded articles not in the English language and preprints. Guidelines only dealing with the definition and/or measurement of blood pressure and screening, and not with pharmacological treatment, were also excluded from the analysis. Regardless of the results of the search, we intentionally analyzed the recommendations of American, European, and local scientific societies related to hypertension, gynecology, and obstetrics. We selected recommendations for this review paper based on their publication date and practical relevance. We have intentionally omitted the recommendations of local scientific societies with only national coverage while analyzing the content of these recommendations for additional relevant information and references. The only local recommendations included in the review were the up-to-date recommendations from the authors’ country. All the authors unanimously accepted the validity of including these specific recommendations due to their high scientific value and coverage.

It should be noted that co-authors of the article (A.P., P.D., and P.K.) are leading co-authors of the recommendations of the Polish Society of Hypertension and Polish Cardiac Society [6] and the Polish Cardiac Society and Polish Society of Gynecologists and Obstetricians [4], which further lent credibility to the selection of the recommendation for this manuscript.

We focus on analyzing the most comprehensive guidelines that discuss the use of antihypertensive drugs during pregnancy and the postpartum period, including: the 2023 European Society of Hypertension (ESH) Guidelines for the Management of Arterial Hypertension [7], the 2024 European Society of Cardiology (ESC) Guidelines for the management of elevated blood pressure and hypertension [8], Guidelines for the management of hypertension in Poland 2024—the position paper of the Polish Society of Hypertension and Polish Cardiac Society experts [6]; Polish Society of Hypertension; Polish Cardiac Society and Polish Society of Gynecologists and Obstetricians on Management of hypertension in pregnancy (PTGiP) [4]; the 2022 International Society for the Study of Hypertension in Pregnancy (ISSHP) Recommendations [9] and the 2023 Society of Obstetric Medicine of Australia and New Zealand (SOMANZ) Hypertension in Pregnancy Guideline [10]; the 2019 American College of Obstetricians and Gynecologists (ACOG) Clinical Guidelines on Gestational Hypertension and Preeclampsia [11] and Chronic Hypertension in Pregnancy [12]; the Society for Maternal-Fetal Medicine (SMFM) statements [13,14,15,16] and also 2019 National Institute for Health and Care Excellence (NICE) Guidelines on Hypertension in pregnancy: diagnosis and management (last updated 17 April 2023) [17]. These recommendations have also been incorporated into the Polish national guidelines for managing hypertension during pregnancy [4], and the position paper of the Polish Society of Hypertension and Polish Cardiac Society experts also sticks strictly to the latter paper.

Additional relevant guidelines of international scope addressing the use of antihypertensive agents during lactation that were identified in the course of preparing this manuscript include the American Academy of Pediatrics (AAP) Policy Statement on Breastfeeding and the Use of Human Milk [18] and the Society of Obstetricians and Gynaecologists of Canada (SOGC) Guideline No. 426: Hypertensive Disorders of Pregnancy [19]. However, these documents do not specifically address antihypertensive therapy in the postnatal period and instead defer to the Drugs and Lactation Database (LactMed) as the primary resource for drug safety information. Consequently, they fall outside the scope of our in-depth analysis.

All six authors (EM, PS, AP, PD, MG, and PK) utilized the Appraisal of Guidelines for Research and Evaluation II (AGREE II) instrument to conduct a quality appraisal of the methodological development of the eight included guidelines (Table 1) [20].

Since LactMed is a database created by recognized experts (Philip O. Anderson and Jason Sauberan), has undergone a very thorough review process, and is also recommended by many guidelines of highly recognized scientific societies, we decided to include this database in our manuscript as one of the essential recommendations [21] while trying to cite the original sources from the literature where possible.

In addition, the authors performed a non-systematic review of manuscripts (without limitation to the type of manuscript and date of publication) on the topic in the following electronic sources: MEDLINE, EMBASE, SCOPUS, and GOOGLE SCHOLAR. We used the following terms in our search: (“HYPERTENSION”) AND (“BREASTFEEDING” OR “POSTPARTUM”) AND (“DRUG” OR “MEDICATION” OR “TREATMENT”) published to May 2025, achieving 1554 records. Additional literature items were obtained based on references in obtained manuscripts, recommendations, and literature from the LACTMED database.

## 3. Pharmacokinetics of Drugs in Breastfeeding

Drugs ingested by a lactating mother pass from maternal plasma into milk by passive diffusion, active transport, lipid co-transport, and transcytosis [22]. The extent of drug transfer is influenced by various physicochemical properties, including the drug’s acid-base profile, its relative protein binding in plasma and milk, lipid solubility, and the composition of the milk itself [23]. Generally, the higher lipid solubility of a drug corresponds to greater concentrations in human milk. Protein binding appears to be one of the strongest determinants of drug passage into breastmilk. Both plasma and breastmilk contain proteins that can bind drugs. The net effect of protein binding is that highly protein-bound drugs tend to remain in the plasma and to pass into the milk in only low concentrations [24].

Unfortunately, population-based evidence is still scarce on the safety of drugs during breastfeeding. In the absence of clinical lactation data, it may be possible to predict the passage of drugs into breast milk (M/P ratio) using only the physicochemical properties of the drug and milk characteristics. Some recently published studies use Physiologically Based Pharmacokinetic (PBPK) modeling to predict drug exposures in mothers and infants. PBPK models were designed to provide information on a drug’s maximum concentration over time, absorption and distribution kinetics, and drug elimination. However, those models have significant limitations and are not yet clinically useful due to the relative lack of consistency in how they are developed and how their quality is assessed [25].

In 2019, the US Food and Drug Administration (FDA) released a guidance document for pharmaceutical companies providing recommendations on how to address the potential impact of maternal drug exposure, including assessment of levels of the drug (and metabolite) appearing in breast milk, the potential effects on breastfeeding infants, and effects of the drug on milk production. It is important to note that high drug levels, increased toxicity, and prolonged therapy may elevate the risk of side effects in a breast-fed infant. Furthermore, newborns and premature infants eliminate drugs at a significantly slower rate than older children due to immature kidney and liver function. As a result, the same medication taken by a breastfeeding mother may have varying effects depending on the age of the breastfed child [26].

Medications taken by breastfeeding mothers often represent a continuation of treatments initiated before or during pregnancy to manage chronic conditions. During pregnancy, drug concentrations in the fetal circulation are generally comparable to maternal therapeutic levels due to placental transfer [22]. In contrast, when pharmacotherapy is continued postpartum and the mother breastfeeds, drug levels in the infant’s serum typically decline over time. This is largely because, although many drugs pass from maternal plasma into breast milk, the amount ingested by the infant is usually low and further reduced by the infant’s ability to metabolize and eliminate the drug [27]. To assess drug intake of the infant, we use Relative Infant Dose (RID)—the percentage of a weight-adjusted infant dose of drug per time relative to the maternal dose over the same time period on a body weight basis [28]. An RID of 100% indicates that the infant dose via milk is equal to a maternal therapeutic dose per weight, while the values of 5–10% are considered safe in infant risk assessment [29]. However, as real-time measurements of drug concentrations in breast milk are rarely available, RID is typically not usable as a practical tool for guiding clinical management.

Risk assessment should consider not only the potential effects of the drug on the infant but also the significant benefits of breastfeeding, the risks posed by untreated maternal conditions, and the mother’s determination to continue breastfeeding.

## 4. Drugs Used to Treat Hypertension During Breastfeeding

Recommendations generally agree that most antihypertensive medications taken by breastfeeding mothers are transferred into breast milk, but usually in very low concentrations [Table 2].

The 2019 PTGiP Recommendations stress that breastfeeding should not be discouraged in women undergoing medical treatment for hypertension. Furthermore, the SOMANZ Guidelines note that there is insufficient evidence to determine whether single-agent therapy is superior to combination therapy for managing postpartum hypertension. Treatment decisions should be made collaboratively through a shared decision-making process involving breastfeeding patients and medical professionals. Antihypertensive drugs recommended for use during breastfeeding across various guidelines include calcium channel blockers, diuretics, alpha-methyldopa, ACE inhibitors, and beta-blockers.

### 4.1. Calcium Channel Blockers

According to the Lactation Database (LactMed), **nifedipine** is excreted into breast milk in small amounts, with no adverse effects noted in infants exposed to it through breastfeeding [30,31]. A study of 21 women taking a median dosage of 40 mg of nifedipine daily shows that the infants would receive an average daily dosage of 0.1% of their mother’s weight-adjusted dosage in breast milk [32]. Furthermore, nifedipine can be used to treat painful nipple vasospasm in nursing mothers who do not respond to other measures [33,34]. The ESH, ESC, PTGiP, NICE, SMFM, and SOMANZ guidelines all consider nifedipine to be safe for use during breastfeeding. Notably, the 2023 SOMANZ Guidelines emphasize that nifedipine is the most extensively studied calcium channel blocker in this setting, with no adverse effects reported in breastfed infants.

Studies on **verapamil** excretion into breast milk show varying results, with the estimated infant dose (adjusted for maternal weight) ranging from 0.01% to 0.5% [35,36]. Verapamil is known to potentially cause hyperprolactinemia and galactorrhea, though the clinical significance of these effects in breastfeeding mothers remains unclear [37,38]. According to LactMed, verapamil is unlikely to cause adverse effects in breastfed infants, particularly those older than two months [39]. However, as antihypertensive treatment is often initiated in the immediate postpartum period, further research is needed to evaluate the safety of verapamil in newborns under two months of age. The guidelines compared in our study provide inconclusive recommendations regarding the use of verapamil. The guidelines compared in our study provide inconclusive recommendations regarding the use of verapamil. The ESH and ESC guidelines consider verapamil safe for use during breastfeeding. However, the PTGiP recommendations emphasize the lack of sufficient safety data on verapamil and advise that, due to its potential adverse effects—such as atrioventricular block; bradycardia; dizziness; headache; persistent constipation; and flushing—it should be reserved for women who do not respond to or cannot tolerate methyldopa; labetalol; or extended-release nifedipine [4].

According to the Lactation Database (LactMed), no relevant published information is available on **diltiazem’s** effects on lactation or breast milk [40]. Based on limited data, the amount of diltiazem ingested by the infant is small and is not expected to cause any adverse effects. According to one published case report, an exclusively breastfed infant would receive a maximum of 0.9% of the maternal weight-adjusted dosage [41]. Therefore, diltiazem is recommended for use during breastfeeding in both the ESC and SOMANZ guidelines.

According to LactMed, limited data suggest that **amlodipine** levels in breast milk are typically low, and plasma levels in breastfed infants are undetectable [42]. Three published case reports of postpartum women taking amlodipine found no physical or neurological abnormalities in the development of their infants [42,43,44]. The PTGiP recommendations indicate that amlodipine can be considered for treating breastfeeding patients if extended-release nifedipine is unavailable. Of note, NICE guidelines emphasize that nifedipine and amlodipine are especially advised for women of Black African or Caribbean family origin [17]. The role of calcium channel blockers in the analyzed guidelines is shown in Table 3.

### 4.2. Diuretics

According to a study by Cominos et al., low doses of **furosemide** (20 mg daily) do not suppress lactation. However, if higher doses are needed, an alternate drug may be preferred [45]. Two randomized, controlled trials comparing postpartum furosemide in low doses (20 mg daily) to placebo in women who had gestational hypertension and preeclampsia found no difference in patient-reported breastfeeding difficulties [46,47]. However, it is important to note that while no statistical significance was observed in either study, the small sample sizes and the absence of a study design specifically focused on breastfeeding outcomes limit the ability to draw definitive conclusions regarding the impact of furosemide on lactation.

Similarly to furosemide, high doses of **hydrochlorothiazide** (above 50 mg daily) may reduce breast milk production [48]. The excretion of hydrochlorothiazide into breast milk appears to be low. One study found that an infant breastfed by a mother taking a 50 mg oral hydrochlorothiazide received approximately 2% of the mother’s weight-adjusted daily dose [49]. Currently, there are no reports of adverse effects on infants from hydrochlorothiazide use during breastfeeding.

**Spironolactone** appears to be safe for use during breastfeeding, as it is poorly excreted into breast milk. One study indicated that a nursing infant would receive about 0.2% of the mother’s total daily dosage [50]. Additionally, it is unlikely that spironolactone alone would be potent enough to suppress lactation [51]. While spironolactone can cause gynecomastia, the risk is very low compared to other drugs [52]. Currently, there are no reported adverse effects associated with spironolactone use during breastfeeding.

The recommendations for hypertension treatment during pregnancy and breastfeeding analyzed in our study are inconsistent regarding the use of diuretics while breastfeeding [Table 4]. The SOMANZ Guidelines state that there is no clear evidence of harm when using diuretics during breastfeeding, and they can be considered when clinically indicated. However, the ESH and PTGiP guidelines caution that certain diuretics may reduce milk production based on the understanding that intense diuresis from high doses of diuretics (especially combined with fluid restriction and breast binding) can negatively affect lactation [53,54]. However, this statement is based on a publication indicating that diuretics may reduce milk production and are generally not the preferred choice for breastfeeding women [55]. Likewise, other studies suggest that high-dose diuretics can negatively impact maternal milk supply [56,57]. Furthermore, another publication cited in the ESC guidelines highlights the lack of sufficient human data on the effects of diuretic use during pregnancy [58]. Otherwise, NICE and ACOG guidelines recommend avoiding diuretics in breastfeeding women whenever possible [12,17].

### 4.3. Alpha-Methyldopa

The amounts of alpha-methyldopa ingested by infants breastfed by mothers taking this medication are low and are not expected to cause any adverse effects. In a case report by Hauser et al., a nursing infant whose mother was taking 250 mg of alpha-methyldopa twice daily showed no detectable levels of methyldopa in the urine, and no adverse effects in the infant were reported [59]. A study involving three women treated with 500 mg doses of alpha-methyldopa during breastfeeding estimated that infants received less than 0.2% of the mother’s total dosage [60].

Currently, to the authors’ knowledge, there have been no reported acute or long-term adverse effects in breastfed infants associated with the use of alpha-methyldopa during breastfeeding. Alpha-methyldopa is consistently recommended as a safe and effective option during breastfeeding across all reviewed guidelines. Nevertheless, the ESH (2023) and ESC (2024) guidelines seem to underscore the heightened risk of postpartum depression linked to its use, advising caution when prescribing this medication to mothers who are at an increased risk of developing depression [Table 5]. In addition, a synthesis of randomized controlled trials and review articles by Wiciński et al. provides epidemiological and pharmacological evidence that methyldopa contributes to postpartum depression, mediated by alterations in hormone levels, reduced cerebral perfusion, and neuronal dysfunction [61]. NICE guidelines recommend stopping methyldopa within 2 days after the birth and changing to an alternative treatment if necessary [17].

### 4.4. Angiotensin-Converting Enzyme (ACE) Inhibitors

Several retrospective studies suggest that ACE inhibitors used during pregnancy may impair fetal kidney development, presumably due to reduced renal blood flow in the fetus [62,63,64]. However, the results remain inconclusive, and to the authors’ knowledge, there have been no proven or reported cases of abnormal kidney development in infants exposed to ACE inhibitors via breast milk. Furthermore, a meta-analysis performed by Walfisch et al. showed that the potential teratogenicity of ACE inhibitors is not associated with first-trimester exposure [65]. To prevent possible teratogenic effects later in pregnancy, women taking ACE inhibitors should be advised to discontinue the medication upon confirmation of pregnancy.

Because of the low levels of **enalapril** in breastmilk, amounts ingested by the infant are small and would not be expected to cause any adverse effects in breastfed infants [66]. According to a study by Redman et al., the estimated maximum intake of an exclusively breastfed infant would be about 0.16% of the maternal weight-adjusted dosage [67]. To the best of the authors’ knowledge, no adverse effects in infants exposed to enalapril through breastfeeding have been reported to date. Regarding maternal adverse effects, a 1989 study by Lombardi et al. involving nine hypertensive postmenopausal women treated with enalapril reported a decrease in plasma protein levels after 15 days of use [68]. However, due to the small sample size and the absence of breastfeeding participants, further research specifically involving lactating women is needed to assess the potential maternal adverse effects of enalapril during breastfeeding.

A study of eleven mothers taking **captopril** while breastfeeding reported that a nursing infant’s maximum daily dosage is less than 0.014% of the mother’s weight-adjusted daily dosage [69]. The data on prolactin levels when using captopril are contradictory. One series of controlled studies with patients having prolactin-secreting tumors showed no effect of captopril on prolactin release [70]. However, in another study, one woman out of 12 subjects was unable to produce enough milk for the study while taking captopril 100 mg three times daily, despite having successfully breastfed for 6 months [9]. It is not known if this decrease was an effect of captopril.

The studies estimate that a breastfed infant would receive about 1.6% of the maternal weight-adjusted dosage of quinapril and 0.14% of the maternal weight-adjusted **benazepril** dosage [23,71]. No adverse outcomes from the use of **quinapril** and benazepril during breastfeeding have been reported thus far.

A study by Chugh et al., which involved five women taking **lisinopril** during breastfeeding, demonstrated that the transfer of the drug into human milk is minimal and that it is unlikely to pose a clinically significant risk to healthy breastfed infants [72]. Although the guidelines we reviewed do not specifically recommend lisinopril as the ACE inhibitor of choice during breastfeeding, current data suggest that it may be considered as a treatment option for hypertension in breastfeeding mothers. However, further research involving larger populations is recommended.

The compared recommendations provide conflicting information regarding the safety of taking ACE inhibitors while breastfeeding [Table 6]. The ESH (2023) guidelines recognize ACE inhibitors as compatible with breastfeeding, particularly for women with underlying cardiovascular or chronic kidney disease. The PTGiP (2019) guidelines consider ACE inhibitors contraindicated during pregnancy as they could affect fetal kidney development. This risk should be carefully considered when prescribing ACE inhibitors for hypertension in breastfeeding women, as ovulation can still occur during lactation, potentially leading to unintended pregnancy [73,74]. According to PTGiP recommendations, enalapril, captopril, and quinapril may be used in breastfeeding women with underlying heart failure or heart disease. Additionally, the ESC guidelines also consider benazepril a safe option. NICE guidelines state that ACE inhibitors are generally not recommended for use by breastfeeding mothers, but they are not absolutely contraindicated. In mothers who are breastfeeding older infants, the use of captopril, enalapril, or quinapril may be considered if an ACE inhibitor is necessary for the mother. Monitoring of the mother’s kidney function and potassium is advised. Adding enalapril to a calcium channel blocker is an option if the latter medication does not control hypertension [17]. According to ACOG guidelines, ACE inhibitors can be used in breastfeeding women [12].

When considering the use of an **ACE inhibitor**, it is important to acknowledge that some studies suggest potential impairment of fetal kidney development when these drugs are used during pregnancy. However, no abnormalities in renal development have been reported in infants exposed to ACE inhibitors via breast milk. Consequently, an individualized assessment of the available evidence is advised. If treatment with an ACE inhibitor is necessary during breastfeeding, enalapril is the preferred option, given its low transfer into breast milk and the lack of reported adverse effects in breastfed infants. Furthermore, enalapril is consistently endorsed as the ACE inhibitor of choice during lactation in clinical guidelines issued by ESC, PTGiP, ISSHP, ACOG, SMFM, and SOMANZ.

### 4.5. Beta-Blockers

Beta-blockers are among the drugs recommended for use by mothers with hypertension during the breastfeeding period. The excretion of beta-adrenergic blocking drugs into breast milk is largely determined by their protein binding. Those with low binding are more extensively excreted into breast milk. Accumulation of the drugs in the infant is related to the fraction excreted in urine [71]. Of note, doses of beta-blockers used in everyday clinical practice often exceed those in lactation studies. Even if RID remains constant, the absolute dose to the infant would increase proportionally with higher maternal doses.

According to LactMed, with 50% protein binding and 5% renal excretion, amounts of **labetalol** ingested by the infant are small and would not be expected to cause any adverse effects in full-term breastfed infants [75]. Reports show that intravenous labetalol can increase serum prolactin. This effect was not shown in women taking labetalol orally [76]. Furthermore, the maternal prolactin level in a mother with established lactation does not affect her ability to breastfeed. Some of the reported side effects in breastfed infants include sinus bradycardia, prolonged QT, and weak sucking [77,78], while the maternal side effects were Raynaud’s phenomenon and a burning sensation of the nipples [79,80]. All side effects associated with the use of labetalol have been reported in isolated cases, and therefore, labetalol is considered safe for use during breastfeeding according to the ESC [8], NICE [17], ACOG [12], and PTGiP [4] guidelines. The SMFM lists labetalol among the preferred drugs but points out situations when the drug is contraindicated (maternal asthma, acute decompensated cardiac function, and bradycardia) [16].

**Metoprolol** is characterized by 10% protein binding, 40% renal excretion, and a moderate half-life, presenting a moderately low risk for accumulation in infants [81]. Thus far, no reports showing maternal adverse effects when using metoprolol during breastfeeding have been found [Table 7]. The SOMANZ Guidelines reference a few reports of infant bradycardia following maternal use of beta-blockers during breastfeeding. However, upon closer examination of the cited cohort study, the findings were not statistically significant, and the specific beta-blocker used by the breastfeeding mothers was not identified [82].

**Propranolol** presents a low risk for accumulation in infants, with 87% protein binding, less than 1% renal excretion, and a moderate half-life [75]. Studies on propranolol use during breastfeeding remain scarce. Some of the reported adverse effects in breastfed infants include sleepiness, bradycardia, and hypoglycemia, but the relevance of this data are questionable [77,82]. Thus far, no adverse reactions in breastfeeding mothers clearly attributable to propranolol were found. However, a case series of mothers with persistent pain associated with breastfeeding shows that propranolol can be used successfully to reduce the symptoms [83].

Similarly, according to LactMed, **atenolol** shows high accumulation in breast milk. In the study by Lwin et al., the mean milk-to-plasma ratio of the patients who were taking 25 to 100 mg of atenolol was 8.57%. In the mother-infant pair study, the ratio (%) of infant plasma drug concentration to maternal plasma drug concentration observed (18.87%) was similar to the relative infant dose estimated (18.20%) [84]. The authors concluded that atenolol use during breastfeeding should be undertaken with some caution. If clinically indicated, an alternate beta blocker may be preferred. Worth noting, a case was described of toxic effects of atenolol consumed during breastfeeding (bradycardia, cyanosis, hypothermia) [85]. Thus, we conclude that if clinically indicated, an alternate beta-blocker may be preferred.

According to LactMed, **nadolol** presents a high risk for accumulation in infants (25% protein binding, 70% renal excretion, and long half-life), and other beta-adrenergic blocking drugs should be chosen [86]. To date, no relevant published information on nadolol effects in breastfed infants has been found. Nadolol is listed among the medications recommended during breastfeeding by the ESC guidelines [8]; however, due to its high protein binding and long half-life, alternative drugs may be preferred, particularly when nursing a newborn or preterm infant.

No relevant published information was found on the adverse effects of **timolol** use when breastfeeding. However, a prospective study on postpartum breastfeeding showed no adverse reactions in the breastfed infants of two mothers taking nadolol [87]. Timolol, with less than 10% protein binding, 20% renal excretion, and a relatively short half-life, presents a moderate risk for accumulation in infants [77]. No relevant published information was found on the adverse effects of timolol use for hypertension during breastfeeding. The only reports are on ophthalmic timolol drops use and showed no adverse effects in breastfed newborns [88,89,90]. There are no mentions of **oxprenolol** use during breastfeeding in LactMed.

LactMed mentions that **nebivolol** and **carvedilol** are characterized by 98% protein binding and a relatively long half-life, presenting a moderate risk for accumulation in infants [91,92]. There is a report of a 2-day-old neonate admitted with persistent severe hypoglycemia and jaundice. The mother reported taking nebivolol 5 mg/day for unspecified tachycardia in the last 4 months of pregnancy. Clinical and instrumental investigations carried out during hospitalization did not reveal any congenital or perinatal abnormalities. After treatment for metabolic and electrolyte imbalance, he was discharged on the 10th day of hospitalization, in good clinical condition and with normalization of clinical and laboratory parameters [93].

The guideline comparison on the use of beta-blockers during breastfeeding is presented in Table 7, and reported side effects in infants and mothers are summarized in Table 8.

### 4.6. Other Drugs Mentioned in the Recommendations

#### 4.6.1. Clonidine

The mechanism of action of **clonidine** is caused by the stimulation of α2-adrenoreceptors of the inhibitory structures of the brain, as well as a reduction of sympathetic impulses to the blood vessels and brain. The hypotensive action of clonidine is associated with a reduction of general peripheral vascular resistance, reduced frequency of cardiac beats, and a reduction of cardiac output [71]. Typical side effects of clonidine include drowsiness, dizziness, constipation, headaches, and xerostomia.

A study from 1987 on 9 nursing women taking oral clonidine shows that an exclusively breastfed infant would receive an estimated 4.1 to 8.4% of the maternal weight-adjusted dosage, which represents an average of about 50% of the simultaneous maternal serum levels [97]. Despite the infant’s high serum levels of clonidine, no typical side effects were reported in this study.

Another study, including an infant breastfed by a mother taking 0.15 mg of clonidine daily, reported drowsiness, hypotonia, suspected generalized seizures, and apnea in the first days of the infant’s life. When breastfeeding was stopped, all symptoms were resolved within 24 h [98]. Furthermore, clonidine has dose-related effects on both oxytocin and prolactin secretion. The effect of the drug on nursing mothers has not yet been well studied, but there is a case report of clonidine-induced postpartum galactorrhea [99].

According to a study by Ornoy A., due to the high serum levels found in some breastfed infants, possible infant side effects, and the potential negative effects on lactation, other antihypertensive agents are preferred, especially while nursing a newborn or preterm infant [100].

Although data on the safety of clonidine during breastfeeding remain conflicting, it is considered a safe option for the treatment of hypertension according to the ESC (2024) and SOMANZ (2023) guidelines [Table 9].

#### 4.6.2. Hydralazine

**Hydralazine** is a vasodilator that is thought to reduce peripheral resistance directly by relaxing the smooth muscle cell layer in arterial vessels. The precise mechanism of action remains unknown but potentially involves an altered Ca^2+^ balance in vascular smooth muscle cells [101]. Some of the more common side effects of hydralazine use include arm, back, or jaw pain; chest discomfort or tightness; irregular heartbeat; reflex tachycardia; and shortness of breath.

Limited milk levels of hydralazine in breastfeeding mothers and its long history of use postpartum indicate that hydralazine is an acceptable antihypertensive for nursing mothers [102]. Furthermore, no adverse effects of hydralazine use during breastfeeding have been reported thus far.

According to ACOG 2019 guidelines, intravenous hydralazine is a first choice in severe intra- or postpartum hypertension [12].

#### 4.6.3. Minoxidil

**Minoxidil** is a widely recognized pharmacological agent commonly used in the management of hair loss and hypertension. Its vasodilatory effect is primarily mediated by the activation of potassium channels in smooth muscle cells, resulting in hyperpolarization of the cell membrane. This hyperpolarization inhibits the influx of calcium ions, crucial for muscle contraction. Consequently, the relaxation and dilation of blood vessels occur, leading to a reduction in blood pressure [101]. Some of the most common side effects of minoxidil use include irregular heartbeat, weight gain, flushing, swelling of the lower legs, and increased hair growth.

According to LactMed, due to the limited information available on the use of oral minoxidil, the drug should be used with caution, especially when the therapy involves a high maternal dosage [103]. Currently, no relevant information has been published on the side effects of oral minoxidil use. However, a case report of a mother using 5% minoxidil topically twice a day during breastfeeding noted the development of considerable black hair on the infant’s forehead at 2 months of age [104].

#### 4.6.4. Angiotensin Receptor Blockers (ARBs)

Some of the most common ARBs used to treat hypertension include **irbesartan** and **losartan**. According to LactMed, neither of those drugs is recommended for breastfeeding mothers due to a lack of information on their use during breastfeeding [105,106].

A recent study by Falconi et al. demonstrates that valsartan, when used in combination with sacubitril by breastfeeding mothers, transfers into human milk at levels below the detection limit [107]. Additionally, two women in the study reported no adverse effects in their breastfed infants. While these findings suggest that valsartan may be considered safe during breastfeeding, further research is needed, as the small sample size limits the ability to draw definitive conclusions.

The ESH 2023 recommendations confirm that ARBs are not recommended in the treatment of hypertension during breastfeeding due to limited safety evidence.

### 4.7. Polypharmacy During Breastfeeding

In the general adult population, starting antihypertensive treatment with a two-drug combination is the cornerstone of BP-lowering therapy in all recommendations [6,7,8]. Starting treatment with a single active substance can only be considered in stage 1 hypertension and low cardiovascular risk [7]. The majority of young postpartum women belong to this group; hence, all the recommendations we have discussed indicate treating this group of patients with a single drug. To the authors’ knowledge, no studies to date have specifically examined the effects of polypharmacy on breastfeeding mothers treated for hypertension. The issue of polytherapy is only addressed in NICE recommendations. British NICE guidelines recommend, in case more than one antihypertensive drug is needed, a combination of a calcium channel blocker with a beta-blocker and/or enalapril [17].

## 5. Summary

A considerable number of breastfeeding mothers require antihypertensive therapy, which is often a continuation of treatment initiated before or during pregnancy. It is well established that most antihypertensive drugs taken by nursing mothers pass into breast milk, though usually in minimal amounts. Therefore, when selecting an appropriate antihypertensive medication during lactation, it is essential to consider all the safety aspects discussed in this article carefully.

**Nifedipine** and **amlodipine** are considered the safest calcium channel blockers for use during breastfeeding, according to all analyzed guidelines and research studies, whereas there are some doubts regarding **diltiazem** and **verapamil**. The guidelines we reviewed offer conflicting advice on **diuretic** use during breastfeeding. Because high doses have been reported to suppress lactation, we recommend prescribing diuretics to lactating patients only at the lowest effective dose to minimize this risk. Given substantial evidence that **alpha-methyldopa** may elevate the risk of postpartum depression, we recommend exercising caution when prescribing it to patients with depression risk factors and considering alternative agents. When considering the use of an **ACE inhibitor**, it is important to acknowledge that so far, no abnormalities in renal development have been reported in infants exposed to these drugs via breast milk. If treatment with an ACE inhibitor is necessary during breastfeeding, enalapril is the preferred option, given its low transfer into breast milk and the lack of reported adverse effects in breastfed infants. Overall, **beta-blockers** with high protein binding and shorter half-lives have a reduced risk of adverse effects during breastfeeding. **Hydralazine** is considered safe for treating hypertension during breastfeeding, with no reported adverse effects. Due to the limited information available on the use of oral **minoxidil**, it should be used with caution. Although some data on **clonidine** remains conflicting, this drug is also considered a safe option. **Angiotensin receptor blockers (ARBs)** are not recommended for the treatment of hypertension during breastfeeding, mainly due to a lack of reliable large-scale data.

It is important to note that for patients using **methyldopa** during pregnancy, the drug should be gradually discontinued after delivery due to the risk of exacerbating depression. In contrast, **labetalol** and **calcium channel blockers** used during pregnancy can be safely continued postpartum. Similarly, if cardioselective **beta-blockers** are indicated, **metoprolol** can be safely used before, during, and after pregnancy. The fact that these medications do not need to be stopped after pregnancy, unlike **methyldopa**, suggests they may be preferred for women who are planning or already pregnant.

Ongoing research and more comprehensive studies are needed to further assess the safety and long-term outcomes of these treatments. Until more conclusive data are available, clinicians should continue to weigh the risks and benefits of antihypertensive therapy on a case-by-case basis for breastfeeding mothers.

## Figures and Tables

**Table 1:** AGREE II scores by guideline.

AGREE II Domain (Scaled % Score)	ESH (2023)	ESC (2024)	PTGiP (2019)	ISSHP (2022)	NICE (2019)	ACOG (2019)	SMFM (2024)	SOMANZ (2023)
Scope and purpose	81.0	82.5	93.7	95.2	92.1	90.5	88.9	92.1
2. Stakeholder involvement	65.1	73.0	95.2	90.5	85.7	79.4	74.6	88.9
3. Rigor of development	80.4	84.5	86.9	89.9	88.1	76.2	72.6	81.5
4. Clarity of presentation	90.5	84.1	95.2	92.1	81.0	85.7	60.3	81.0
5. Applicability	89.3	85.7	83.3	81.0	89.3	79.8	76.2	80.0
6. Editorial independence	90.5	92.9	81.0	92.9	69.0	76.2	81.0	85.7
Overall quality	90.5	88.1	95.2	97.6	83.3	85.7	71.4	88.1

ACOG: American College of Obstetricians and Gynecologists; AGREE: Appraisal of Guidelines, Research and Evaluation; ISSHP: International Society for the Study of Hypertension in Pregnancy; ESH: European Society of Hypertension; ESC: European Society of Cardiology; NICE: National Institute for Health and Care Excellence; PTGiP: Polish Society of Gynecologists and Obstetricians; SMFM: Society for Maternal-Fetal Medicine; SOMANZ: Society of Obstetric Medicine Australian and New Zealand.

**Table 2:** Safety of antihypertensive drugs during breastfeeding, according to compared recommendations.

ESH (2023)	ESC (2024)	PTGiP (2019)	ISSHP (2022)	NICE (2019)	ACOG (2019)	SMFM (2024)	SOMANZ (2023)
Antihypertensive drugs taken by the nursing mother are excreted into breast milk, mostly in very low concentrations.	All blood pressure-lowering drugs are excreted into breast milk. Except for propranolol, atenolol, acebutolol, and nifedipine, most drugs are excreted in very low concentrations in breast milk.	Breastfeeding should not be discouraged in women with hypertension, including those on medical treatment. Although most antihypertensive drugs pass into human breast milk, their concentrations are usually much lower than in serum. Detailed information on the safety of medications in breastfeeding women (including their concentration in breast milk and infantile blood, as well as possible and reported adverse effects) can be found in the LactMed database.	Most antihypertensive agents are acceptable for use in breastfeeding. Up-to-date information can be obtained in LactMed.	The need to take antihypertensive medications should not prevent women from breastfeeding. Consider monitoring the blood pressure of babies, especially those born preterm, who have symptoms of low blood pressure for the first few weeks. When discharged home, advise women to monitor their babies for drowsiness, lethargy, pallor, cold peripheries, or poor feeding.	Antihypertensives, in general, can be used in breastfeeding women. Most antihypertensive medications are detectable, albeit at low concentrations, in breast milk; thus, their use during lactation is not contraindicated.	Many medications used to treat hypertension do not have robust data surrounding their use in breastfeeding. Long-term use of certain medications should be avoided, but they may be appropriate to use in a life-threatening emergency.	Data on the breast milk transmission of the most commonly used agents remains sparse. There remains inadequate data to suggest the superiority of a single agent or group of agents in selecting antihypertensives for the management of hypertension in the postpartum period. The choice of antihypertensive (beta-blockers, methyldopa, hydralazine, nifedipine, enalapril, or clonidine) should be made through a shared decision-making process, particularly in breastfeeding or lactating women.

ACOG: American College of Obstetricians and Gynecologists; ISSHP: International Society for the Study of Hypertension in Pregnancy; ESH: European Society of Hypertension; ESC: European Society of Cardiology; NICE: National Institute for Health and Care Excellence; PTGiP: Polish Society of Gynecologists and Obstetricians; SMFM: Society for Maternal-Fetal Medicine; SOMANZ: Society of Obstetric Medicine Australian and New Zealand; BP: blood pressure; HT: hypertension.

**Table 3:** Comparison of the guidelines for the use of calcium channel blockers during breastfeeding.

ESH (2023)	ESC (2024)	PTGiP (2019)	ISSHP (2022)	NICE (2019)	ACOG (2019)	SMFM (2024)	SOMANZ (2023)
Considered compatible with breastfeeding: nifedipine, verapamil.	Considered safe with breastfeeding: diltiazem, nifedipine, and verapamil.	Extended-release nifedipine: allowed in breastfeeding women Amlodipine: no data on the safety in breastfeeding women. Seems a reasonable choice if extended-release nifedipine is unavailable Verapamil: contradictory data on safety.	No information.	Considered safe with breastfeeding: amlodipine, nifedipine. Calcium channel blockers are especially recommended for women of Black African or Caribbean family origin.	Calcium channel blockers can be used safely during breastfeeding	Nifedipine (extended release) is listed as one of the preferred medications in lactation	Commonly used calcium channel blockers in the postpartum period include nifedipine, amlodipine, and occasionally, diltiazem. Nifedipine: most extensively investigated In this setting, with published safety information suggesting the absence of infant adverse effects with the use of nifedipine in the lactating mother. Passes into breast milk in very small amounts.

ACOG: American College of Obstetricians and Gynecologists; ISSHP: International Society for the Study of Hypertension in Pregnancy; ESH: European Society of Hypertension; ESC: European Society of Cardiology; PTGiP: Polish Society of Gynecologists and Obstetricians; NICE: National Institute for Health and Care Excellence; SMFM: Society for Maternal-Fetal Medicine; SOMANZ: Society of Obstetric Medicine Australian and New Zealand; BP: blood pressure; HT: hypertension.

**Table 4:** Comparison of the guidelines for the use of diuretics during breastfeeding.

ESH (2023)	ESC (2024)	PTGiP (2019)	ISSHP (2022)	NICE (2019)	ACOG (2019)	SMFM (2024)	SOMANZ (2023)
Not contraindicated. They may be associated with reduced milk production.	Considered safe with breastfeeding. Recommended: furosemide, hydrochlorothiazide, spironolactone.	Diuretics should not be used in breastfeeding women as they suppress lactation.	No information.	Where possible, diuretics should be avoided in breastfeeding women	Although the concentration of diuretics in breast milk is low, these agents may reduce the quantity of milk produced.	Hydrochlorothiazide is listed among preferred medications in lactation; however, it was indicated that it could decrease milk production	Diuretics reduce the rate of persistent postpartum hypertension, with no obvious evidenceof harm. Given the limitation in the data, there is not enough evidence to support the routine use of diuretics in women with preeclampsia in the postpartum period. The use of loop diuretics can be considered when there are clinical indications for their use.

ACOG: American College of Obstetricians and Gynecologists; ISSHP: International Society for the Study of Hypertension in Pregnancy; ESH: European Society of Hypertension; ESC: European Society of Cardiology; PTGiP: Polish Society of Gynecologists and Obstetricians; NICE: National Institute for Health and Care Excellence; SMFM: Society for Maternal-Fetal Medicine; SOMANZ: Society of Obstetric Medicine Australian and New Zealand; BP: blood pressure; HT: hypertension.

**Table 5:** Comparison of the guidelines for the use of alpha-methyldopa during breastfeeding.

ESH (2023)	ESC (2024)	PTGiP (2019)	ISSHP (2022)	NICE (2019)	ACOG (2019)	SMFM (2024)	SOMANZ (2023)
Compatible with breastfeeding. Not a drug of first choice because it increases the risk of postpartum depression.	Considered safe with breastfeeding.	Passes to human breast milk in small amounts. It may trigger or exacerbate postpartum depression, sedation, and orthostatic hypotension.	Concerns that methyldopa might increase the risk of postnatal mental health problems are unsubstantiated.	Withdrawing the medication within 2 days after the birth and changing to an alternative treatment if necessary is recommended.	Methyldopa, should be avoided because it can be associated with depression.	Listed among preferred medications in pregnancy, not in lactation.	There remains a paucity of data on adverse effects of methyldopa exposure in infants through breastmilk.

ACOG: American College of Obstetricians and Gynecologists; ISSHP: International Society for the Study of Hypertension in Pregnancy; ESH: European Society of Hypertension; ESC: European Society of Cardiology; PTGiP: Polish Society of Gynecologists and Obstetricians; NICE: National Institute for Health and Care Excellence; SMFM: Society for Maternal-Fetal Medicine; SOMANZ: Society of Obstetric Medicine Australian and New Zealand; BP: blood pressure; HT: hypertension.

**Table 6:** Comparison of the guidelines for the use of ACE inhibitors during breastfeeding.

ESH (2023)	ESC (2024)	PTGiP (2019)	ISSHP (2022)	NICE (2019)	ACOG (2019)	SMFM (2024)	SOMANZ (2023)
Compatible with breastfeeding. It can be used in women with underlying cardiovascular disease or chronic kidney disease.	Considered safe with breastfeeding: benazepril, captopril, enalapril, quinapril.	Contraindicated in pregnancy, but as they pass to human breast milk in negligible amounts, some of them are approved for the treatment (enalapril, captopril, quinapril). Contraindicated in women who breastfeed preterm infants and infants with suspected kidney disease. There are special indications for using ACEi in breastfeeding women with heart failure and peripartum cardiomyopathy.	ACE inhibitors, including captopril, enalapril, and quinapril, are acceptable for use in breastfeeding.	ACE inhibitors are generally not recommended but are not absolutely contraindicated.	Angiotensin-converting enzyme inhibitors (e.g., enalapril and captopril) concentrations in breast milk are low, and these drugs may be used safely during breastfeeding unless high doses arerequired.	Enalapril, captopril, and benazepril are listed among preferred medications in lactation, but close follow-up of the infant’s weight and counsel on a contraceptive plan are recommended	There is a theoretical concern that ACE inhibitors could affect infant kidney development, particularly in infants with extreme prematurity. However, this remains inadequately investigated. Enalapril: Milk levels were undetectable. Data on infant adverse events remain sparse.

ACOG: American College of Obstetricians and Gynecologists; ISSHP: International Society for the Study of Hypertension in Pregnancy; ESH: European Society of Hypertension; ESC: European Society of Cardiology; PTGiP: Polish Society of Gynecologists and Obstetricians; NICE: National Institute for Health and Care Excellence; SMFM: Society for Maternal-Fetal Medicine; SOMANZ: Society of Obstetric Medicine Australian and New Zealand; BP: blood pressure; HT: hypertension.

**Table 7:** Comparison of the guidelines for the use of beta-blockers during breastfeeding.

ESH (2023)	ESC (2024)	PTGiP (2019)	ISSHP (2022)	NICE (2019)	ACOG (2019)	SMFM (2024)	SOMANZ (2023)
No information.	Considered safe with breastfeeding: labetalol, metoprolol, nadolol, oxprenolol, propranolol, and timolol.	Passes to human breast milk in small amounts, although there are significant differences between the individual agents in this drug class. Metoprolol and labetalol are approved for use in breastfeeding women. Newer beta-blockers (nebivolol) and newer drugs with the mechanism of action identical to the one of labetalol (carvedilol) cannot be currently recommended in breastfeeding women due to lack of data.	No information.	Atenolol or labetalol can be added to other drugs if blood pressure is not controlled.	Propranolol and labetalol are preferred for treatment in breastfeeding women due to their high plasma protein binding and low breast milk concentration	Labetalol is listed among preferred medications in lactation	Labetalol: moderately low risk for accumulation in infants, no reported infant adverse events. Metoprolol: moderately low risk for accumulation in infants, While there have been a few case reports of infant bradycardia, there has not been a statistically significant difference in the rate of infant adverse events. Propranolol: a low risk for accumulation in infants. There remains a significant paucity in the literature on any infant adverse events with the use of Propranolol.

ACOG: American College of Obstetricians and Gynecologists; ISSHP: International Society for the Study of Hypertension in Pregnancy; ESH: European Society of Hypertension; ESC: European Society of Cardiology; PTGiP: Polish Society of Gynecologists and Obstetricians; NICE: National Institute for Health and Care Excellence; SMFM: Society for Maternal-Fetal Medicine; SOMANZ: Society of Obstetric Medicine Australian and New Zealand; BP: blood pressure; HT: hypertension.

**Table 8:** Side effects of beta-blocker use during breastfeeding.

Drug	Relative Infant Doses	Reported Adverse Effects in Breastfed Infants	Reported Adverse Effects in Breastfeeding Mothers
LABETALOL	The average dose received by breastfed infants is estimated to be between 0.004% and 0.07% of the maternal weight-adjusted dose [23].	Case Report: Sinus bradycardia: a 26-week premature infant whose mother was taking 300 mg of labetalol twice daily [94]. Case Report: Prolonged QT: a 2-month-old infant whose mother was taking 100 mg of labetalol twice daily [78]. Prospective study: Weak sucking: unreported dosage of labetalol [77].	Intravenous labetalol can increase serum prolactin, and oral labetalol does not increase serum prolactin [81]. Case Report: Raynaud’s phenomenon of the nipples: a woman with a history of symptoms of Raynaud’s phenomenon, 100 mg of labetalol twice daily during breastfeeding after two pregnancies [80]. Case Report: Burning sensation of the nipples: intravenous labetalol for pre-eclampsia [79].
METOPROLOL	At a dose of 50–100 mg daily, the average dose received by the breastfed infants is estimated to range from 0.005% to 0.01% of the maternal weight-adjusted dose [23].	Cohort Study: Of 6 mothers taking metoprolol, none reported adverse effects in her breastfed infant [95]. Prospective Cohort Study: Of 2 mothers taking metoprolol, none reported adverse effects in her breastfed infant [77].	No relevant published information was found.
PROPRANOLOL	A fully breastfed infant would receive between <0.1 and 0.9% of the weight-adjusted maternal dosage of propranolol [23].	Prospective cohort study: of 8 mothers taking propranolol, one reported sleepiness in her breastfed infant. However, the interpretation of this finding is limited, as the mother was concurrently using other unspecified antihypertensive medications that could have contributed to the observed effect [82]. Case report: a case of bradycardia in a 2-day-old infant breastfed by a mother taking propranolol. It is not clear whether the mother had been taking propranolol near birth term and might have transmitted the drug to the infant transplacentally [96]. Prospective cohort study: of 16 mothers taking propranolol while breastfeeding, three women reported their infants’ hypoglycemia, and one reported the infant’s bradycardia [77].	No relevant published information was found.
NADOLOL	It is estimated that a fully breastfed infant would receive about 5.1% of the maternal weight-adjusted dosage of nadolol [23].	No relevant published information was found.	No relevant published information was found.
TIMOLOL	It was estimated that a fully breastfed infant would receive between 0.96% and 1.2% of the maternal weight-adjusted dosage [88].	No relevant published information was found.	No relevant published information was found.
NEBIVOLOL	No relevant published information was found.	No relevant published information was found.	No relevant published information was found.
CARVEDILOL	No relevant published information was found.	No relevant published information was found.	No relevant published information was found.

**Table 9:** Comparison of the guidelines for the use of other drugs during breastfeeding.

ESH (2023)	ESC (2024)	PTGiP (2019)	ISSHP (2022)	NICE (2019)	ACOG (2019)	SMFM (2024)	SOMANZ (2023)
ARBs are not currently recommended (limited safety evidence).	Considered safe with breastfeeding: clonidine, hydralazine, and minoxidil.	No information	No information	Avoiding ARBs is recommended.	Intravenous hydralazine: a first choice in severe postpartum hypertension.	Hydralazine is listed among preferred medications in lactation.	Hydralazine: lack of infant adverse effects reported in the literature.

ACOG: American College of Obstetricians and Gynecologists; ISSHP: International Society for the Study of Hypertension in Pregnancy; ESH: European Society of Hypertension; ESC: European Society of Cardiology; PTGiP: Polish Society of Gynecologists and Obstetricians; NICE: National Institute for Health and Care Excellence; SMFM: Society for Maternal-Fetal Medicine; SOMANZ: Society of Obstetric Medicine Australian and New Zealand; BP: blood pressure; HT: hypertension.

## Data Availability

All data relevant to this review are included in the article.
